# Information seeking and credibility-related cues in Chinese consumers’ organic food purchase intention: an integrated TPB–alphabet model

**DOI:** 10.3389/fnut.2026.1919012

**Published:** 2026-09-25

**Authors:** Yihan Chen, Rosly Othman, Chong Ye, Hui Chen

**Affiliations:** 1 Graduate School of Business, Universiti Sains Malaysia, Penang, Malaysia; 2 School of Economics and Management, Fuzhou University, Fuzhou, China; 3 Department of Mechanical Engineering, Faculty of Engineering, University of Malaya, Kuala Lumpur, Malaysia

**Keywords:** credibility-related cues, food choice, information seeking, organic food, purchase intention, TPB–alphabet model

## Abstract

**Introduction:**

In China’s rapidly expanding organic food market, organic food remains a credence-based food category in which consumers rely on information and certification cues to evaluate product authenticity, safety, and sustainability. This study examines how information seeking and organic food credibility cues shape Chinese consumers’ organic food purchase intention by integrating the Theory of Planned Behavior (TPB) with selected components of Alphabet Theory.

**Methods:**

Data were collected from 412 screened consumers in mainland China and analyzed using partial least squares structural equation modeling.

**Results:**

Information seeking was positively associated with organic food knowledge, which in turn was positively associated with attitude toward organic food. Attitude, subjective norm, perceived behavioral control, social media, eco-labels, and perceived government support all positively influenced purchase intention. Bootstrapped indirect-effect analysis further confirmed a sequential pathway from information seeking to purchase intention through knowledge and attitude.

**Discussion:**

The findings indicate that organic food choice in China is formed through both internal learning-evaluation processes and external credibility-related cues operating at digital, product-certification, and institutional levels. The study contributes to food consumer science by explaining how consumers interpret organic food claims under information asymmetry and offers implications for food retailers, certification bodies, and policymakers.

## 1 Introduction

The organic food market has expanded steadily worldwide, yet consumer adoption remains uneven across countries and regions. China represents a particularly important case. Although the market has grown rapidly in recent years and has become one of the world’s largest organic food markets in terms of sales, its penetration rate remains low compared with mature markets (1). Organic food is also a credence-based food category because consumers cannot easily verify production methods, certification validity or environmental claims before purchase. Therefore, the central challenge is not only supply growth but also the formation of stable consumer purchase intention under information asymmetry and trust uncertainty.

In China, this challenge is intensified by several market-specific conditions. Organic food typically carries a substantial price premium, certification systems are not always fully trusted by consumers, and product-related information is increasingly mediated by digital platforms (2). At the same time, government policies, traceability initiatives and eco-label systems are becoming more visible in the marketplace (3, 4). These developments suggest that purchase intention in the Chinese organic food context is shaped not only by personal evaluations but also by the information and credibility cues through which consumers assess organic food claims.

The Theory of Planned Behavior (TPB) has been widely applied to explain organic food purchase intention through attitude, subjective norm, and perceived behavioral control (5–7). Extended TPB studies have added health consciousness, environmental awareness, trust, and other contextual factors, but often treat attitude as an already formed antecedent rather than explaining the information-learning process through which it develops. Alphabet Theory complements TPB by emphasizing information seeking and knowledge formation (8). Two prior TPB-Alphabet applications provide the closest precedents. One treated revealed information and knowledge as parallel antecedents of attitude among Chinese consumers (9). The other linked information seeking to product knowledge in Indonesian social-commerce food chains, with perceived sustainability preceding attitude (10). These studies establish the relevance of information and knowledge, but do not directly test the specific serial pathway from information seeking to purchase intention through knowledge and attitude in China’s organic-food market. The present study addresses this gap by specifying this learning-evaluation pathway within the TPB intention model.

A second limitation concerns the fragmented treatment of external influences in existing organic food research. Prior studies have separately examined the effects of social media, eco-labels, certification information, and government-related factors on consumer purchase decisions (3, 4, 11, 12). Thus, the issue is not that these factors have been overlooked, but that they have generally been examined as separate predictors and from different theoretical perspectives. Social media is commonly treated as a communication or information-exposure factor, eco-labels as product- or certification-level signals, and government support as a policy or regulatory factor. To provide a more coherent explanation, this study conceptualizes these factors as credibility-related cues operating at three levels: social media as a digital information cue, eco-labels as product-level certification cues, and government support as an institutional assurance cue.

This layered perspective is particularly relevant to China, where organic food consumption takes place within a market characterized by rapid digitalization and an increasingly formalized certification and regulatory system. As shown in Figure 1, China has progressively strengthened its organic food governance through certification standards, labeling requirements, traceability initiatives, and market supervision from 1994 to 2026. These developments make institutional assurance especially relevant alongside digital information and product-level certification cues. Thus, the Chinese organic food market provides an appropriate context for examining how consumers use multiple credibility-related cues when evaluating difficult-to-verify organic food claims.

**Figure 1:**
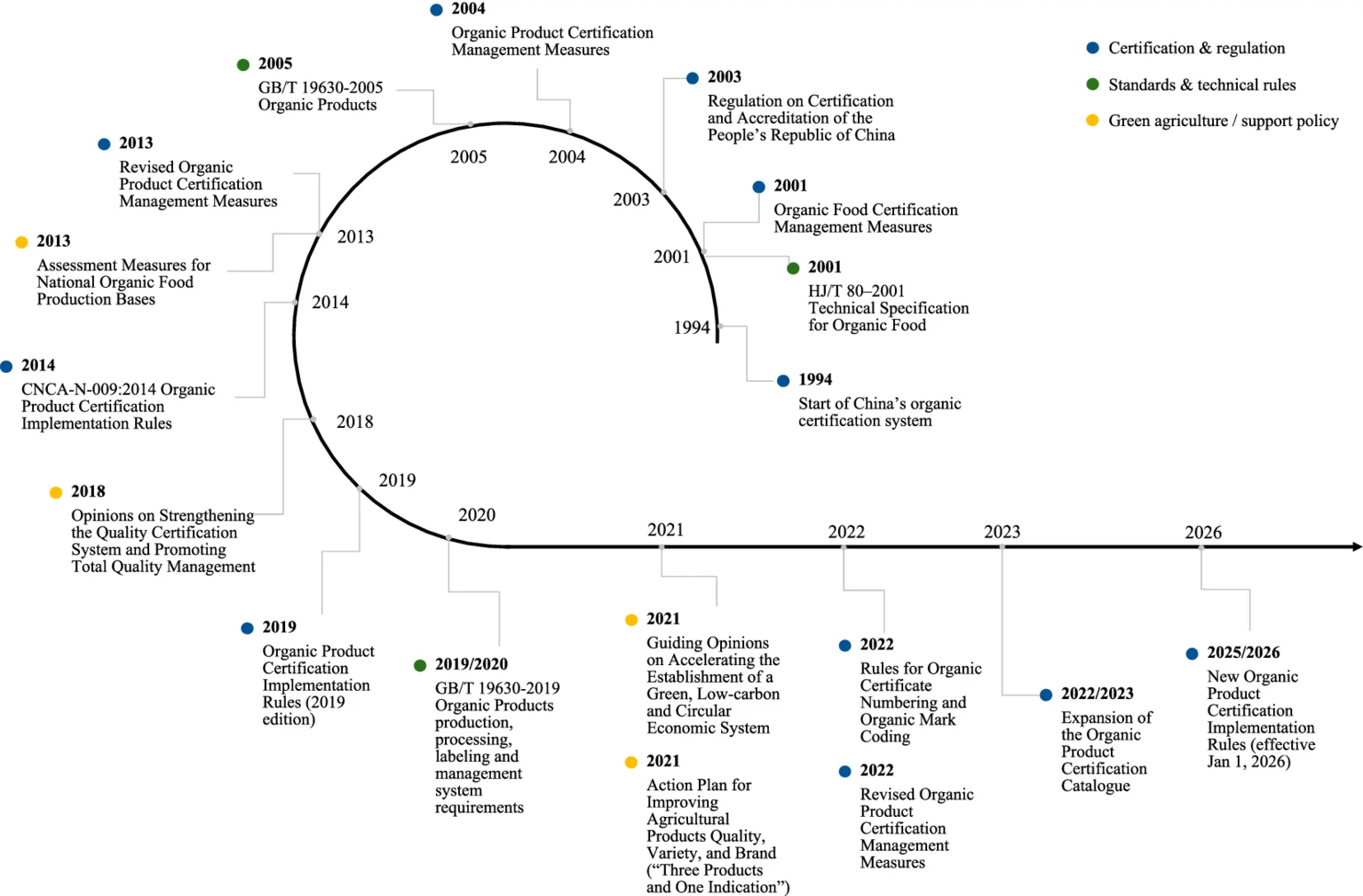
Evolution of China’s organic certification and regulatory framework, 1994–2026.

To address these two limitations, this study develops an integrated framework combining selected components of Alphabet Theory with TPB (10). The model introduces an upstream cognitive pathway in which information seeking contributes to knowledge formation and knowledge subsequently shapes attitude. Attitude, subjective norm, and perceived behavioral control then represent the core TPB predictors of purchase intention. At the same time, social media, eco-labels, and government support capture the digital, product-certification, and institutional credibility environment surrounding organic food consumption. The framework therefore connects the internal learning-evaluation process through which consumers form judgments with the external credibility cues through which they assess organic food claims. It also allows the sequential indirect pathway from information seeking to purchase intention through knowledge and attitude to be examined.

This study makes three contributions. First, it provides a more process-oriented extension of TPB by incorporating the information-seeking and knowledge-formation mechanism emphasized in Alphabet Theory, thereby explaining how attitude can develop before influencing purchase intention. Second, it conceptually organizes social media, eco-labels, and government support as a layered system of credibility-related cues operating at digital, product-certification, and institutional levels, rather than treating them as unrelated additional predictors. Third, it connects the internal learning-evaluation process with the external information and assurance environment in a single model, thereby offering a dual-domain explanation of purchase intention in a credence-based food market.

Accordingly, this study aims to explain how information seeking, organic food knowledge, core TPB predictors, and external credibility-related cues jointly shape organic food purchase intention among sampled consumers in mainland China.

## 2 Theoretical background and hypothesis development

### 2.1 Theoretical foundation

Studies on organic food consumption have frequently utilized the Theory of Planned Behavior (TPB) to explain purchase intention (5, 6, 13). TPB proposes that behavioral intention is shaped by three immediate antecedents: attitude, subjective norm and perceived behavioral control (14). Attitude reflects consumers’ favorable or unfavorable evaluation of purchasing organic food, subjective norm captures perceived social pressure from important others, and perceived behavioral control refers to consumers’ perceived ability to perform the purchase behavior (14). This framework has been widely applied because it captures the evaluative, normative and control-based considerations that shape consumers’ intentions. Nonetheless, the Theory of Planned Behavior (TPB) is inadequate in elucidating the initial establishment of attitudes, particularly in information-intensive and trust-sensitive markets like organic food (12).

To address this limitation, the present study draws on Alphabet Theory. Unlike TPB, Alphabet Theory highlights the cognitive pathway through which consumers search for information, accumulate knowledge and develop evaluative judgments (8). In the organic food context, consumers often cannot directly verify production methods, certification validity or environmental benefits at the point of purchase. They therefore rely on information about labels, standards, production processes, health benefits and environmental claims to form knowledge and evaluate product credibility (15). In this study, Alphabet Theory is used to explain how information seeking contributes to knowledge formation, which subsequently shapes consumers’ attitudes toward organic food.

The integration of TPB and Alphabet Theory serves a clear analytical purpose. Alphabet Theory explains the upstream cognitive process through which information seeking and knowledge shape attitude (8), whereas TPB explains how attitude, subjective norm and perceived behavioral control translate into purchase intention (14). Building on this TPB-Alphabet integration, this study further incorporates social media, eco-label and government support as external credibility-related cues. Social media is conceptualized as a digital information cue because it increases consumers’ exposure to food-related information, product recommendations and user-generated evaluations through platform-mediated communication (16). Eco-label is conceptualized as a product-level certification cue. In the organic food context, eco-label refers to visible environmental or certification-related labels that help consumers infer organic authenticity, environmental compliance and product credibility (17). Government support is conceptualized as an institutional assurance cue because it indicates regulatory oversight, policy backing and market supervision (18). This extension is theoretically relevant because organic food purchasing in China is shaped by information asymmetry and trust uncertainty. Since consumers cannot directly verify production methods, certification validity or environmental claims before purchase, they rely on external cues to assess the reliability of organic food claims. Together, social media, eco-label and government support complement TPB by capturing the external information and credibility environment in which Chinese consumers form purchase judgments.

Based on this theoretical integration, this study develops an integrated research framework combining alphabet theory and the extended theory of planned behavior. Information seeking and knowledge represent the upstream cognitive pathway emphasized by Alphabet Theory, while attitude, subjective norm and perceived behavioral control form the core psychological structure of TPB. Social media, eco-label and government support are incorporated as external credibility-related cues to capture the external information and credibility environment of China’s organic food market. By connecting these elements, the framework explains how consumers move from information acquisition and knowledge formation to purchase intention. Figure 2 presents the proposed research framework and the hypothesized relationships among the constructs.

**Figure 2:**
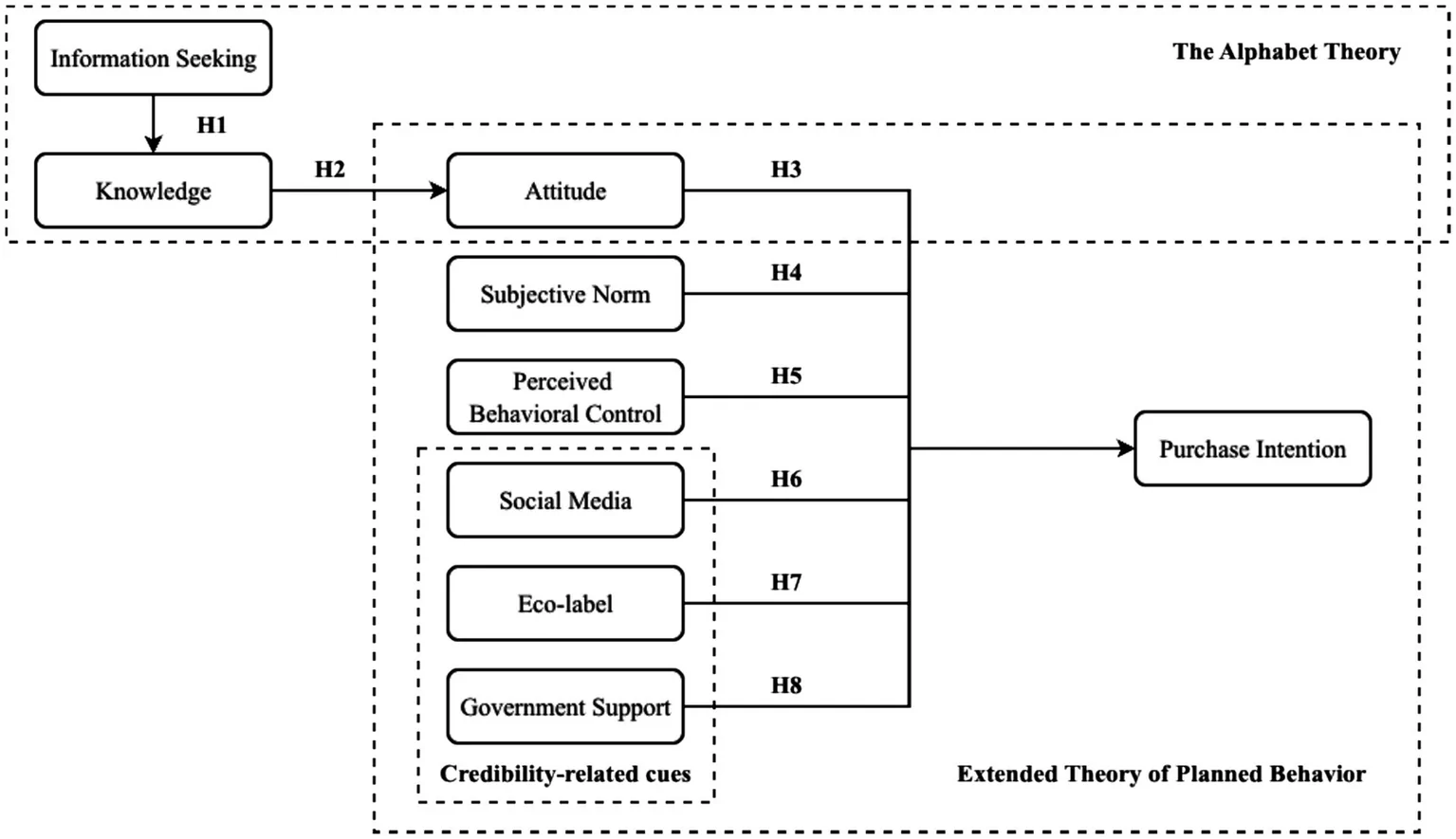
Proposed research framework integrating alphabet theory and the extended theory of planned behavior.

### 2.2 Hypothesis development

#### 2.2.1 Information seeking, knowledge, and attitude

Information seeking refers to consumers’ active efforts to acquire product-related information from different sources, including websites, search engines, social media, videos and online communities (19). In the present study, information seeking is conceptualized primarily as an active information-acquisition behavior, reflecting the extent to which consumers deliberately search for organic-food-related information. In organic food markets, information seeking is particularly important because many product attributes, such as production standards, certification credibility, environmental value and health-related benefits, cannot be directly verified at the point of purchase (15, 20). Consumers therefore rely on external information to reduce uncertainty and develop a clearer understanding of organic food (21, 22).

Conceptually, knowledge refers to consumers’ understanding of organic food attributes, certification standards, quality cues and consumption value (23). In the present study, however, the adopted self-report scale captures a broader knowledge-related orientation, including both perceived acquired knowledge and consumers’ preference for obtaining sufficient understanding of organic food before purchase. Rather than being a fixed personal trait, knowledge can be developed through repeated information exposure, active search, prior experience and social interaction (9, 10). From the perspective of Alphabet Theory, information seeking provides the cognitive input through which consumers learn what organic food is, how it differs from conventional food and why certification or labeling matters (8, 24). Previous studies have also suggested that consumers who actively seek product information tend to develop higher levels of product knowledge (10, 25, 26). Therefore, consumers who actively search for organic food information are expected to develop stronger knowledge of organic food. Based on this, this paper proposes the following hypothesis:

*H1*: Information seeking positively influences knowledge of organic food.

Knowledge is also an important antecedent of consumers’ attitudes toward organic food. When consumers have a stronger understanding of organic food, they are better able to evaluate its health benefits, environmental value, certification standards and quality attributes (23, 27). Such knowledge can reduce ambiguity, increase perceived credibility and strengthen favorable evaluations of organic food (9, 28). In line with Alphabet Theory, knowledge helps transform information exposure into more stable evaluative judgments (8, 10, 29). Previous studies indicate that organic food knowledge is positively associated with consumers’ attitudes toward organic food (24, 30, 31). Accordingly, this study proposes the following hypothesis:

*H2*: Knowledge positively influences attitude toward organic food.

#### 2.2.2 TPB predictors and purchase intention

Attitude is one of the central predictors of behavioral intention in the Theory of Planned Behavior (14). It reflects consumers’ favorable or unfavorable evaluation of purchasing organic food. When consumers perceive organic food as healthier, safer, more environmentally responsible and more valuable than conventional alternatives, they are more likely to form a positive attitude toward its purchase (32). Within TPB, such favorable evaluations increase the desirability of choosing organic food relative to conventional alternatives and thereby strengthen consumers’ motivation to purchase. Prior studies on green and organic consumption have consistently shown that attitude is an important determinant of purchase intention (33–35). In the organic food context, a favorable attitude may increase consumers’ perceived value of organic products and strengthen their willingness to purchase them (36). Therefore, this study proposes the following hypothesis:

*H3*: Attitude positively influences purchase intention toward organic food.

Subjective norm refers to the perceived social pressure from important others, such as family members, friends, peers and social groups (14). In food consumption decisions, individuals may be influenced by the expectations, opinions and behaviors of people around them. Such perceived normative pressure can be particularly relevant in organic food consumption because organic purchasing is often associated with health awareness, environmental responsibility and socially desirable consumption (37). When consumers perceive that important others approve of or expect organic food purchasing, they may be more motivated to form a purchase intention. Although prior studies have not consistently supported the effect of subjective norm on purchase intention (38), perceived normative pressure remains theoretically relevant and empirically supported in organic and sustainable consumption contexts (5, 39, 40). Accordingly, this study proposes the following hypothesis:

*H4*: Subjective norm positively influences purchase intention toward organic food.

Perceived behavioral control refers to the perceived ease or difficulty of performing a behavior (14). In organic food purchasing, this construct captures consumers’ judgments about whether they have sufficient resources, access and ability to identify reliable organic products. Organic food is often associated with higher prices, limited availability and information complexity (6, 41). As a result, even favorable attitudes may not translate into intention if consumers perceive organic food purchasing as difficult. Prior studies have also shown that perceived behavioral control is an important predictor of green and organic food purchase intention (42, 43). When consumers perceive greater control over buying organic food, their purchase intention is expected to increase. Therefore, this study proposes the following hypothesis:

*H5*: Perceived behavioral control positively influences purchase intention toward organic food.

#### 2.2.3 Credibility-related cues and purchase intention

Beyond individual cognition and TPB predictors, organic food purchase intention may also be shaped by external credibility-related cues. In this study, social media, eco-label and government support are conceptualized as three credibility-related cues operating at different levels: social media as a digital information cue, eco-label as a product-level certification cue and government support as an institutional assurance cue. Together, these cues complement TPB by capturing the external information and credibility environment in which consumers form purchase intention. Their relevance is particularly pronounced in organic food markets, where consumers cannot directly verify many product attributes and therefore depend on external signals to reduce information asymmetry and uncertainty.

Social media has become a major channel through which consumers encounter food-related information, product recommendations and user-generated evaluations (16). In China, organic food information is increasingly disseminated through digital platforms, including short videos, live-streaming, online reviews, influencers and peer-generated content. In this study, social media is conceptualized as a platform-mediated digital information cue, focusing on the information environment through which consumers access and encounter organic-food information. Its role lies in reducing the effort required to access product information and exposing consumers to product claims, peer experiences and user-generated evaluations. For a credence-based product such as organic food, these digital cues can help consumers interpret otherwise difficult-to-verify product attributes and reduce uncertainty surrounding the purchase decision. However, social media exposure does not necessarily strengthen purchase intention by itself; its positive influence is more likely when the information encountered is perceived as relevant and sufficiently credible to support consumers’ evaluation of organic food claims (44). Prior studies have also suggested that social media information can influence consumers’ green and organic consumption (45–47). Therefore, this study proposes the following hypothesis:

*H6*: Social media positively influences purchase intention toward organic food.

Eco-labels provide visible signals about environmental standards, product authenticity and certification compliance (17). In organic food markets, labels are especially important because many product attributes cannot be directly verified by consumers at the point of purchase (48). In the present study, eco-labeling is treated as a credibility-related certification cue rather than as a direct measure of perceived label credibility. From a credibility-cue perspective, eco-labels function as product-level certification cues that help consumers infer whether organic food claims are reliable. By translating otherwise unobservable production and environmental attributes into a visible certification signal, eco-labels can reduce information asymmetry and strengthen consumers’ confidence in product authenticity. However, this signaling function depends on consumers being able to recognize and trust the label; an unfamiliar or distrusted label provides limited information for evaluating product credibility. In China, where trust in organic certification remains uneven, eco-labels may therefore provide an important product-level cue for purchase evaluation (3). Prior studies have similarly shown that eco-labels and certification-related labels can positively influence green and organic food purchase intention (11, 49, 50). Therefore, this study proposes the following hypothesis:

*H7*: Eco-label positively influences purchase intention toward organic food.

Government support reflects consumers’ perceptions that public institutions provide regulation, policy backing and market supervision for organic food development (18). In this study, government support is conceptualized as an institutional assurance cue. Such institutional assurance is important in organic food markets because consumers often rely on public authority to assess credibility, market order and regulatory protection. When consumers cannot independently verify whether organic certification standards are effectively enforced, perceived government support can serve as a third-party assurance mechanism by signaling regulatory oversight, certification supervision and protection against misleading market claims. Such institutional assurance can reduce uncertainty about market governance and strengthen confidence in the credibility of organic products. However, this mechanism is more likely to operate when consumers perceive government regulation and support as credible and effectively implemented rather than merely symbolic. In China, where organic food development is closely linked to certification supervision, food safety regulation and sustainable consumption policies, this institutional assurance may be particularly relevant to purchase decisions. Prior research similarly suggests that government policies and institutional support can encourage pro-environmental and sustainable consumption behavior (4, 18, 51). Therefore, this study proposes the following hypothesis:

*H8*: Government support positively influences purchase intention toward organic food.

## 3 Materials and methods

### 3.1 Research design and sample

This study adopted a quantitative, cross-sectional survey design to examine the factors associated with organic food purchase intention among Chinese consumers. A survey design was appropriate because the study aimed to measure consumers’ perceptions and test theoretically derived relationships among multiple latent constructs.

The target population consisted of adult consumers in mainland China who had purchased organic food within the previous 3 months. Purposive sampling was adopted because the study required respondents who were able to meaningfully evaluate organic food information, eco-labels, government support and purchase-related perceptions. The purpose was not to obtain a nationally representative sample of all Chinese consumers, but to recruit a theoretically relevant consumer segment for testing the proposed model. The sample size adequacy was evaluated with G*Power. With a medium effect size of 0.15, an alpha level of 0.05, and a statistical power of 0.80, the minimum necessary sample size was 98. The total sample of 412 valid responses surpassed the minimum criteria and was deemed sufficient for PLS-SEM analysis.

### 3.2 Measurement development and questionnaire design

To ensure measurement consistency and empirical rigor, a structured, closed-ended questionnaire comprising three primary sections was developed based on established scales from prior studies. The first section included the informed consent statement and screening questions. Participants were informed about the study’s purpose, the voluntary aspect of their involvement, and the utilization of their responses for research purposes. Screening questions were employed to confirm that respondents were over 18 years old, lived in mainland China and had purchased organic food during the previous 3 months. The second section measured the model variables using previously validated items adapted from the literature, including information seeking, knowledge, attitude, subjective norm, perceived behavioral control, social media, eco-label, government support and purchase intention. The constructs, item numbers, measurement sources and roles in the model are summarized in Table 1. The full measurement items are provided in Supplementary Appendix 1. All construct items were assessed on a five-point Likert scale, with values ranging from 1 = strongly disagree to 5 = strongly agree. The third section gathered demographic data from respondents, encompassing gender, age, marital status, education, and monthly income.

**Table 1:** Measurement constructs and sources.

Construct	No. of items	Sources	Role in the model
Information seeking	3	Sadeli et al. (10)	Upstream cognitive antecedent
Knowledge	3	Sadeli et al. (10)	Cognitive learning construct
Attitude	3	Xing et al. (41)	TPB predictor
Subjective norm	3	Xing et al. (41)	TPB predictor
Perceived behavioral control	3	Xing et al. (41)	TPB predictor
Social media	4	Chi (65)	Digital information cue
Eco-label	3	Alam et al. (11)	Product-level certification cue
Government support	2	Ding et al. (66)	Institutional assurance cue
Purchase intention	4	Xing et al. (41)	Outcome construct

The constructs were operationalized in accordance with their theoretical roles and the measurement scales adopted from prior studies. The established TPB-related constructs, including attitude, subjective norm, perceived behavioral control, and purchase intention, were measured using items that reflect their conventional conceptual meanings. The eco-label measure captures familiarity with eco-labeling, recognition of the label, and the environmental information conveyed through the label, rather than perceived label credibility itself. Government support was likewise operationalized as perceived institutional support and regulatory assurance. Because information seeking, social media, and knowledge involve related information-oriented processes, their operational distinctions warrant further clarification. Information seeking is operationalized primarily as an active information-acquisition behavior, reflecting the extent to which consumers deliberately search for organic-food-related information. Social media is operationalized as a platform-mediated digital information cue, focusing on the information environment through which consumers access and encounter organic-food information. The adopted knowledge measure is a self-report scale that captures a broader knowledge-related orientation, including both perceived acquired knowledge and consumers’ preference for obtaining sufficient understanding of organic food before purchase.

### 3.3 Data collection

Data were collected in China through an online self-administered questionnaire distributed via Wenjuanxing between December 2025 and January 2026. Respondents were recruited through Wenjuanxing’s online respondent pool and through survey-link distribution on commonly used Chinese social-media platforms, including WeChat, RedNote, and Weibo. A participation incentive was provided to respondents who completed the survey. These channels were used for respondent recruitment and data collection, while the sampling strategy remained purposive sampling based on the predefined eligibility criteria.

Before completing the questionnaire, respondents were informed of the purpose of the study, the anonymous nature of participation and their right to withdraw at any time. Participation was voluntary, and no personally identifiable information was collected. To ensure sample suitability, only respondents aged 18 years and above and with prior organic food purchase experience were invited to complete the survey. The questionnaire was developed in both English and Chinese, and the Chinese version was refined through a back-translation procedure. Before the main survey, a pre-test with 10 eligible respondents was conducted to assess wording clarity, layout, readability and completion time. A pilot test with 30 consumers was then conducted to assess preliminary reliability and further refine the instrument.

A total of 463 submitted questionnaires were obtained after the initial eligibility screening. The returned responses were subsequently subjected to additional data-quality checks using platform-recorded information and the exported response data. Specifically, responses were examined for duplicate submissions, unusually short completion times, highly repetitive response patterns across the main measurement items, and logically inconsistent responses. Based on these quality checks, 51 questionnaires were excluded, leaving 412 valid responses for the final analysis, corresponding to a usable response rate of 89.0%. No missing values were observed because all substantive questions were mandatory in the online survey.

### 3.4 Data analysis

Partial least squares structural equation modeling (PLS-SEM) was employed to test the proposed TPB–Alphabet research model. PLS-SEM was considered appropriate because this study aimed to examine theoretically specified relationships among multiple latent constructs and explain variance in key endogenous constructs, including organic food knowledge, attitude toward organic food and purchase intention (52). The data were analyzed using SmartPLS 4.1.

Following the two-step approach, the measurement model was first assessed by examining outer loadings, Cronbach’s alpha, composite reliability, average variance extracted and the heterotrait-monotrait ratio (52, 53). The structural model was then evaluated by examining inner VIF values, path coefficients, coefficient of determination, effect sizes, predictive relevance and bootstrapped confidence intervals (52). Bootstrapping with 5,000 subsamples was used to assess the significance of direct effects, specific indirect effects and the serial indirect effect from information seeking to purchase intention through knowledge and attitude. Because all constructs were measured using the same self-administered questionnaire, potential common method bias was addressed through both procedural and statistical approaches. Procedurally, participation was anonymous and voluntary, previously validated measures were used, and back-translation, pre-testing, and pilot testing were conducted to improve item clarity and reduce ambiguity. Statistically, common method bias was assessed using Harman’s single-factor test and a full-collinearity assessment.

## 4 Results

### 4.1 Respondents’ demographic profile

In terms of demographic characteristics, the sample was slightly female-dominated, with 56.6% female respondents and 43.4% male respondents. The respondents were also relatively young, with the largest proportion aged 25–34 years (47.3%), followed by those aged 35–44 years (25.7%). Most respondents were married (58.0%). The educational profile was relatively high, with 45.4% holding a bachelor’s degree and 20.4% holding a master’s degree or above. In addition, 59.7% reported monthly income of at least RMB 10,000. Geographically, respondents were drawn from all seven major regions of mainland China, with East China accounting for the largest share (31.6%), followed by South China (18.2%) and North China (15.8%). Table 2 presents the comprehensive demographic profile of the respondents. Overall, the sample captured a theoretically relevant consumer segment with relatively high education, digital exposure and purchasing power, making it suitable for examining organic food purchase intention in China.

**Table 2:** Respondents’ demographic profile.

Demographic	Category	Frequency	Percentage
Gender	Male	179	43.4%
	Female	233	56.6%
Age	18–24 years old	39	9.5%
	25–34 years old	195	47.3%
	35–44 years old	106	25.7%
	45–54 years old	58	14.1%
	55 years old and above	14	3.4%
Marital status	Single	155	37.6%
	Married	239	58.0%
	Divorced	18	4.4%
Education	High school or below	55	13.3%
	Associate degree	86	20.9%
	Bachelor’s degree	187	45.4%
	Master’s degree or above	84	20.4%
Monthly income (RMB)	Less than 5,000	60	14.6%
	5,000–10,000	106	25.7%
	10,000–15,000	117	28.4%
	15,000–20,000	75	18.2%
	More than 20,000	54	13.1%
Region	North China	65	15.8%
	Northeast China	24	5.8%
	East China	130	31.6%
	Central China	57	13.8%
	South China	75	18.2%
	Southwest China	43	10.4%
	Northwest China	18	4.4%

### 4.2 Common method bias assessment

Before assessing the measurement model, common method bias was examined using Harman’s single-factor test because all constructs were measured using the same self-administered questionnaire (54). All measurement items were entered into an unrotated exploratory factor analysis. The results showed that the first unrotated factor accounted for 38.502% of the total variance (see Supplementary Appendix 2), which was below the commonly used threshold of 50% (54). In addition, no single dominant factor emerged. These results suggest that common method bias was unlikely to be a serious concern in this study.

Because all constructs were collected from the same respondents using a self-administered questionnaire, common method bias was assessed using complementary statistical diagnostics. First, Harman’s single-factor test showed that the first unrotated factor accounted for 38.502% of the total variance, below the commonly used threshold of 50% (54), and no single dominant factor emerged (see Supplementary Appendix 2). In addition, a full-collinearity assessment was conducted for all latent constructs following Kock (55). The full-collinearity VIF values ranged from 1.521 to 2.310, with all values below the recommended threshold of 3.3 (see Supplementary Appendix 3). Taken together, these results suggest that common method variance was unlikely to materially distort the estimated relationships in the model.

### 4.3 Measurement model assessment

The measurement model was assessed using outer loadings, Cronbach’s alpha, composite reliability (CR) and average variance extracted (AVE). As shown in Table 3, all outer loadings exceeded 0.708, ranging from 0.724 to 0.909, indicating satisfactory indicator reliability. Cronbach’s alpha and composite reliability values were above 0.70, and all AVE values exceeded 0.50, supporting internal consistency reliability and convergent validity.

**Table 3:** Reliability and convergent validity.

Constructs	Items	Outer loadings	CR	AVE	Cronbach’s alpha
Attitude(ATT)	ATT1	0.857	0.904	0.759	0.841
	ATT2	0.873			
	ATT3	0.884			
Eco-label(EL)	EL1	0.830	0.881	0.711	0.797
	EL2	0.854			
	EL3	0.847			
Government support(GS)	GS1	0.890	0.892	0.804	0.757
	GS2	0.904			
Information seeking(IS)	IS1	0.872	0.889	0.728	0.813
	IS2	0.849			
	IS3	0.837			
Knowledge(KN)	KN1	0.873	0.903	0.756	0.839
	KN2	0.872			
	KN3	0.862			
Perceived behavioral control(PBC)	PBC1	0.856	0.879	0.707	0.793
	PBC2	0.839			
	PBC3	0.827			
Purchase intention(PI)	PI1	0.895	0.889	0.669	0.833
	PI2	0.862			
	PI3	0.724			
	PI4	0.780			
Social media(SM)	SM1	0.889	0.932	0.775	0.903
	SM2	0.889			
	SM3	0.909			
	SM4	0.832			
Subjective norm (SN)	SN1	0.845	0.873	0.696	0.782
	SN2	0.859			
	SN3	0.798			

This study applied the HTMT criterion, which has been recommended as a more sensitive and stringent assessment of discriminant validity in PLS-SEM (53). HTMT values should remain well below 1.0, with 0.90 commonly used as the upper threshold and 0.85 regarded as a more conservative criterion (56, 57). In the present study, all HTMT values were below 0.85, with the highest value at 0.753, thereby providing further evidence of discriminant validity (see Table 4). Taken together, these findings indicate that the measurement model was satisfactory and suitable for the subsequent structural model assessment.

**Table 4:** Heterotrait-monotrait ratio (HTMT) criterion.

Construct	ATT	EL	GS	IS	KN	PBC	PI	SM	SN
ATT									
EL	0.697								
GS	0.481	0.691							
IS	0.532	0.432	0.416						
KN	0.753	0.548	0.401	0.752					
PBC	0.601	0.712	0.577	0.567	0.629				
PI	0.611	0.737	0.671	0.399	0.494	0.703			
SM	0.446	0.640	0.509	0.364	0.423	0.739	0.643		
SN	0.516	0.602	0.483	0.322	0.372	0.544	0.657	0.435	

### 4.4 Structural model assessment

Before testing the structural paths, collinearity among predictor constructs was assessed. The inner VIF values ranged from 1.419 to 2.155, which were below the recommended threshold of 3.3, indicating that collinearity among predictor constructs was not a serious concern. The significance of the direct paths was then assessed using bootstrapping with 5,000 resamples.

As shown in Table 5, all hypothesized direct effects were statistically significant. Information seeking had a significant positive relationship with knowledge (*β* = 0.625, *p* < 0.001), supporting H1. Knowledge also had a significant positive relationship with attitude (*β* = 0.635, *p* < 0.001), supporting H2. For the direct predictors of purchase intention, attitude (*β* = 0.128, *p* = 0.002), subjective norm (*β* = 0.210, *p* < 0.001), perceived behavioral control (*β* = 0.137, *p* < 0.001), social media (*β* = 0.191, *p* < 0.001), eco-label (*β* = 0.149, *p* = 0.005), and government support (*β* = 0.183, *p* < 0.001) all had significant positive relationship with purchase intention. Among these direct predictors of purchase intention, subjective norm showed the largest path coefficient, followed by social media and government support. Therefore, H1 to H8 were supported. The overall structural model, including standardized path coefficients and R^2^ values for the endogenous constructs, is presented in Figure 3.

**Table 5:** Path coefficients analysis.

Hypotheses	Path	β	STDEV	*t*-value	*p*-value	CI 95%		Decision
						LL	UL	
H_1_	IS → KN	0.625	0.033	18.929	<0.001	0.560	0.688	Supported
H_2_	KN → ATT	0.635	0.032	19.863	<0.001	0.569	0.694	Supported
H_3_	ATT → PI	0.128	0.040	3.159	0.002	0.047	0.205	Supported
H_4_	SN → PI	0.210	0.038	5.535	<0.001	0.135	0.282	Supported
H_5_	PBC → PI	0.137	0.039	3.490	<0.001	0.055	0.212	Supported
H_6_	SM → PI	0.191	0.040	4.741	<0.001	0.115	0.272	Supported
H_7_	EL → PI	0.149	0.053	2.837	0.005	0.047	0.254	Supported
H_8_	GS → PI	0.183	0.047	3.912	<0.001	0.092	0.274	Supported

**Figure 3:**
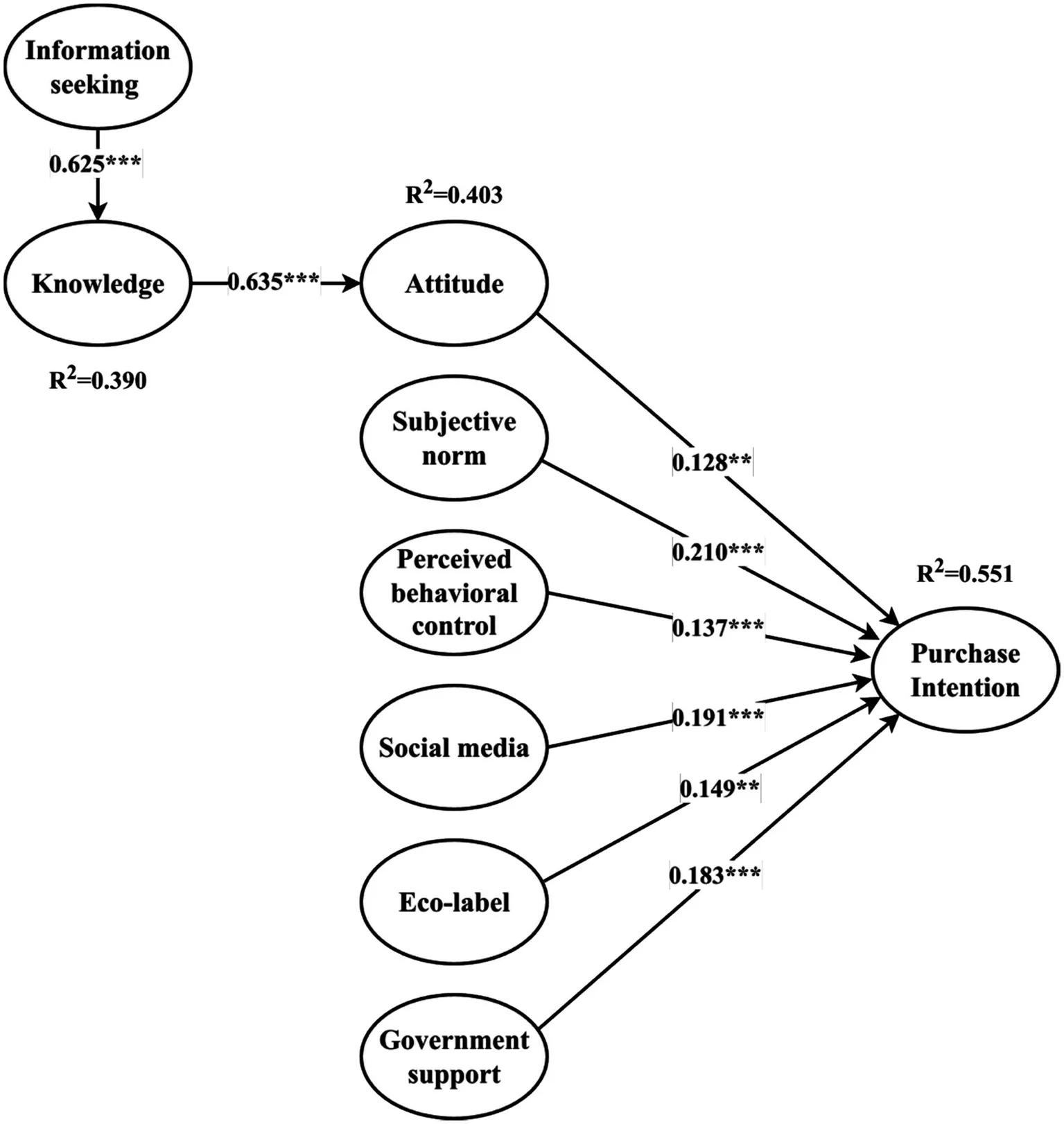
Path coefficients of the structural model. Standardized path coefficients are reported. **p < 0.01; ***p < 0.001.

Given the sequential structure of the model, specific indirect effects were further assessed using bootstrapping. As shown in Table 6, the indirect effect of information seeking on attitude through knowledge was significant (*β* = 0.397, *p* < 0.001), with a confidence interval excluding zero. The indirect effect of knowledge on purchase intention via attitude was also significant (*β* = 0.081, *p* = 0.002). More importantly, the serial indirect effect of information seeking on purchase intention through knowledge and attitude was significant (*β* = 0.051, *p* = 0.002), supporting the proposed sequential indirect pathway.

**Table 6:** Specific indirect effects.

Indirect effect	β	STDEV	*t*-value	*p*-value	95% CI		Decision
					LL	UL	
IS→ KN → ATT	0.397	0.031	12.758	<0.001	0.337	0.458	Supported
KN → ATT → PI	0.081	0.026	3.141	0.002	0.030	0.132	Supported
IS→ KN → ATT → PI	0.051	0.016	3.072	0.002	0.019	0.083	Supported

The explanatory power and predictive relevance of the structural model were evaluated using *R*^2^, adjusted *R*^2^ and *Q*^2^-values. As shown in Table 7, the *R*^2^-values for knowledge, attitude and purchase intention were 0.390, 0.403 and 0.551, respectively, indicating moderate explanatory power. The adjusted *R*^2^-values were close to the corresponding *R*^2^-values, indicating stable explanatory estimates. In addition, all *Q*^2^-values were greater than zero, supporting predictive relevance for all endogenous constructs. The *f*^2^ results further showed large effects for IS → KN (*f*^2^ = 0.640) and KN → ATT (*f*^2^ = 0.675), while the direct predictors of purchase intention showed small effects ranging from 0.021 to 0.069, as shown in Table 8. This indicates that the information–knowledge–attitude pathway was particularly important, whereas purchase intention was jointly explained by several psychological and credibility-related cues rather than by a single dominant predictor.

**Table 7:** Structural model *R*^2^ and *Q*^2^.

Endogenous construct	*R* ^2^	Adjusted *R*^2^	*Q* ^2^
Knowledge	0.390	0.389	0.291
Attitude	0.403	0.402	0.302
Purchase intention	0.551	0.544	0.356

**Table 8:** Effect sizes (*f*^2^) of the latent variables.

Path	*f* ^2^	Effect size
ATT → PI	0.022	Small
EL → PI	0.023	Small
GS → PI	0.050	Small
IS → KN	0.640	Large
KN → ATT	0.675	Large
PBC → PI	0.021	Small
SM → PI	0.044	Small
SN → PI	0.069	Small

## 5 Discussion

This study examined organic food purchase intention among surveyed consumers in China using an integrated behavioral framework. The framework combines selected components of Alphabet Theory with TPB and incorporates social media, eco-label, and government support as context-specific predictors. By integrating an information-based cognitive pathway with core TPB constructs and external credibility-related cues, the study provides a more comprehensive explanation of the relationships underlying purchase intention within the surveyed consumer sample. The discussion below interprets these findings from cognitive, behavioral and contextual perspectives.

This study confirms a significant positive relationship between information seeking and knowledge, and between knowledge and attitude toward organic food (H1, H2). More importantly, the two results should be read as a connected cognitive pathway rather than as two isolated effects. Specifically, the findings are consistent with a sequential association in which more active information seeking is associated with a stronger knowledge-related orientation, including greater perceived acquired knowledge and a stronger preference for obtaining sufficient understanding of organic food before purchase. This knowledge-related orientation, in turn, is positively associated with consumers’ attitudes toward organic food.

This interpretation is consistent with recent evidence from Bazhan et al. (30), who found that organic-food knowledge contributes to purchase intention indirectly through attitude, and with earlier research emphasizing the role of product knowledge in strengthening favorable evaluations of organic food (29, 35, 40). It also extends recent TPB-based studies examining how health and environmental motivations operate through the core TPB constructs (2, 5). Whereas these studies focus on how consumption motivations are translated into intention through attitude, subjective norm, and perceived behavioral control, the present study examines an earlier stage of the decision process by identifying information seeking and knowledge as upstream antecedents of attitude. The significant serial indirect effect from information seeking to purchase intention through knowledge and attitude further supports this learning-evaluation interpretation.

The findings support the continued relevance of attitude, subjective norm and perceived behavioral control to organic food purchase intention (H3, H4, H5), although their relative effects differed. This pattern broadly converges with recent TPB-based organic-food research. Xing and Liao (2) showed that health and environmental motivations influence organic-food purchasing through attitude and subjective norm, while Li and Shan (5) reported significant roles for attitude, subjective norm and perceived behavioral control in green-packaged organic-food purchase intention. Bazhan et al. (30) likewise identified attitude and subjective norm as important pathways to organic-food purchase intention. However, the present study differs in the relative importance of these TPB components: subjective norm showed a stronger association with purchase intention than attitude or perceived behavioral control within the surveyed sample. This finding is particularly noteworthy because it suggests that, within this consumer segment, personal evaluation operates alongside a comparatively salient normative influence rather than constituting the dominant route to intention. Attitude nevertheless remained significant, indicating that consumers’ own evaluations continue to matter, while the positive effect of perceived behavioral control highlights the continuing importance of affordability, accessibility and purchasing feasibility. This interpretation also remains consistent with earlier TPB-based organic-food research emphasizing the relevance of these three behavioral antecedents (58, 59).

The significant effects of social media, eco-label and government support (H6, H7, H8) further indicate that purchase intention within the surveyed sample is associated not only with individual cognition and TPB-related factors, but also with external credibility-related cues. Among these cues, social media showed the strongest association with purchase intention. This finding is consistent with recent social-commerce research showing that ratings and reviews were associated with organic-food purchase intention, while social influence and influencer endorsements were associated with both purchase intention and social-commerce trust (60). It also accords with Bhagat et al. (44), who reported that food-vlogger credibility and information quality were associated with consumers’ attitudes and purchase intentions. However, Samaniego-Arias et al. (61) found no direct relationship between social media and organic-product purchase intention; instead, social media operated through attitude, subjective norm, and perceived behavioral control. This divergence may reflect differences in platform context and construct operationalization: some studies treat social media as an upstream influence on TPB evaluations, whereas the present model estimates its informational role alongside those evaluations. Together, these findings indicate that digital communication may influence organic-product decisions through both direct and indirect routes, depending on the type of platform information and the psychological mechanisms represented in the model. The present result therefore supports the conceptual distinction between social media as a platform-mediated information cue and subjective norm as interpersonal normative pressure. Beyond digital information cues, the eco-label and government-support findings also align with recent trust-based research (62). Yang et al. (63) found that information acquisition ability was associated with eco-label trust both directly and indirectly through label knowledge and institutional trust among consumers in China. This complements earlier organic-food research showing that certification and labeling cues can reduce uncertainty and support purchase evaluation (3, 48). The present results extend this trust-building perspective by showing that both product-level certification cues and perceived institutional assurance are positively associated with purchase intention. The comparatively smaller effect of eco-label may indicate that the presence of a label alone is insufficient when consumers have difficulty recognizing or interpreting it, whereas the significant government-support effect is consistent with prior evidence linking confidence in government regulation with organic-food consumption (18, 64). Thus, these findings support a layered credibility structure in which social media functions as a digital information cue, eco-labels as product-level certification cues, and government support as an institutional assurance cue.

Overall, the findings position the present study alongside recent research that increasingly moves beyond single-factor explanations of organic food consumption. Whereas recent studies have separately emphasized TPB-based motivations, digital and social-commerce influences, or certification trust, the present framework connects these domains by integrating an upstream learning-evaluation pathway with TPB predictors and credibility-related cues. Within the surveyed sample, purchase intention was therefore associated with the combined roles of cognitive learning, normative influence, perceived feasibility, digital information, product-level certification and institutional assurance.

## 6 Implications and limitations

### 6.1 Theoretical implications

This study makes several theoretical contributions to the literature on organic food consumption and consumer purchase intention. First, it extends the conventional TPB framework by showing that attitude should not be treated solely as an exogenous antecedent of purchase intention. Instead, the results support the relevance of an upstream cognitive pathway linking information seeking with knowledge and knowledge with evaluative judgment. By integrating selected components of Alphabet Theory into the TPB framework, the study offers a more process-oriented explanation of how purchase intention is formed in the organic food context.

Second, this study refines the treatment of external influences in organic food purchase intention research by introducing a layered view of external credibility-related cues. Rather than adding contextual variables in a broad manner, the study distinguishes three external cues operating at different levels: social media as a digital information cue, eco-label as a product-level certification cue and government support as an institutional assurance cue. This distinction clarifies the theoretical relevance of these external signals to an extended TPB model, demonstrating how they support consumers’ purchase intention under information asymmetry and trust uncertainty in China’s organic food market.

Third, the study contributes to the growing literature on organic food consumption in emerging markets, especially China. Much of the existing research has relied on TPB to explain organic food intention, but has paid less attention to the roles of information asymmetry, trust uncertainty and policy presence. By incorporating eco-label and government support alongside social media, the present study provides a more context-sensitive framework for explaining purchase intention in credibility-sensitive food markets. In this sense, the study not only broadens the explanatory scope of TPB, but also demonstrates the value of integrating cognitive and contextual variables into intention models.

### 6.2 Practical implications

The practical value of the findings lies less in identifying a single dominant lever than in showing that organic food purchase intention is associated with consumer learning, social influence, market assurance, and perceived feasibility. Because the direct predictors produced individually small effect sizes, interventions should be coordinated across communication, certification, regulation, and market access rather than implemented in isolation.

An effective strategy should begin by improving the quality and usability of consumer information. The information seeking-knowledge-attitude pathway indicates that communication should help consumers convert exposure into usable knowledge rather than merely increase message frequency. Producers, farmer cooperatives, and retailers could provide concise, verifiable explanations of production practices, certification requirements, and differences from conventional food through packaging, traceability links, and digital content. The roles of social media and subjective norm further suggest combining credible online communication with peer recommendations, farm visits, and community education. Universities and research institutions could verify scientific claims, while consumer associations and civil society organizations could translate technical information and correct misleading content.

These communication efforts should be reinforced by a verification system that enables consumers to assess organic claims independently. The findings for eco-labels and perceived government support point to the value of a shared assurance system linking certification bodies, regulators, producers, and consumers. Official labels could be presented consistently and linked through QR codes to a unified public platform showing certificate validity, certification scope, production entities, and traceability records. Public agencies could publish accessible information on inspections, violations, corrective actions, and certificate suspensions, while maintaining clear complaint and recall channels. Risk-based inspections and technical assistance for small producers and cooperatives could strengthen compliance, and independent organizations could assess whether certification information is understandable to different consumer groups.

These communication and assurance measures should be accompanied by improvements in affordability and access. The positive roles of attitude and perceived behavioral control suggest combining value communication with smaller package sizes, clearer explanations of price premiums, shared logistics, direct-to-consumer channels, community pickup points, and better integration of online and offline retail. Overall, the findings support a shared-responsibility model. Producers and cooperatives can improve transparency, retailers and platforms can improve access, and certification bodies and regulators can strengthen verification. Universities, research institutions, consumer associations, and civil society organizations can contribute independent education and evaluation.

### 6.3 Limitations and future research

Despite its contributions, this study has several limitations that should be acknowledged. First, the study adopted a cross-sectional design, which limits the ability to draw stronger causal inferences about how consumers move from information seeking to knowledge, attitude and purchase intention over time. Although the proposed relationships are theoretically grounded, future research could use longitudinal or experimental designs to examine whether these effects remain stable across different stages of consumer decision-making.

Second, the study focused on purchase intention rather than actual purchase behavior. This means that the findings explain how intention is formed, but do not directly capture whether intention is translated into real purchasing outcomes. Future studies could therefore extend the present framework by incorporating actual behavioral data, repurchase behavior or intention–behavior consistency in order to provide a fuller account of organic food consumption.

Third, the purposive and demographically concentrated sample limits the generalizability of the findings. Respondents were relatively young, highly educated, and higher-income, and the sample was not nationally representative. Therefore, the findings should be interpreted as reflecting the surveyed consumer segment rather than Chinese consumers as a whole. Future research should employ more representative sampling across regions and demographic groups and test the model in other national contexts.

Finally, although the present study incorporated key cognitive, behavioral and contextual variables, other potentially relevant factors were not included in the model. Future research may consider extending the framework by examining variables such as price sensitivity, trust in certification, traceability perception, health consciousness or product availability in order to further refine the explanation of organic food purchase intention.

## 7 Conclusion

This study examined the determinants of organic food purchase intention among Chinese consumers by integrating the Theory of Planned Behavior with Alphabet Theory. Using survey data from 412 valid respondents and analyzing the model through PLS-SEM, the study found that information seeking was positively associated with knowledge, knowledge was positively associated with attitude, and attitude, subjective norm, perceived behavioral control, social media, eco-label, and government support all positively influence purchase intention. These findings show that purchase intention is not driven by TPB variables alone. It is also linked to upstream information-related learning and to external credibility-related cues that help consumers interpret organic food claims under information asymmetry and trust uncertainty.

The study contributes to the literature by clarifying how attitude is formed, by offering a more coherent integration of TPB and Alphabet Theory, and by contextualizing organic food intention in China’s distinctive market environment. It also offers practical guidance for market actors, certification bodies, policymakers, and other stakeholders seeking to expand organic food consumption through stronger information design, better communication strategies, more credible labeling, and visible regulatory support. In sum, the study provides a more complete explanation of intention formation in an emerging organic market and lays the groundwork for future research on consumer decision-making in other trust-sensitive food domains.

## Data Availability

The original contributions presented in the study are included in the article/Supplementary material, further inquiries can be directed to the corresponding authors.
