# Supplementary material for: Information seeking and credibility-related cues in Chinese consumers’ organic food purchase intention: an integrated TPB–alphabet model
Source: Front Nutr. 2026 Sep 25;13:1919012. doi: 10.3389/fnut.2026.1919012 (PMC13649402; doi:10.3389/fnut.2026.1919012)
Supplement: Supplementary file 1 [file Supplementary_file_1.docx]

# Appendix 1

Questionnaire

| **Variable** | **Item** | **Statement** |
| --- | --- | --- |
| Information Seeking (IS) | IS1 | I use social networking sites to seek information related to organic food. |
|  | IS2 | I read or take part in forums or chat groups online to seek information related to organic food. |
|  | IS3 | I watch videos online to seek information related to organic food. |
| Knowledge (KN) | KN1 | I have gained much knowledge about organic food. |
|  | KN2 | I want to have a deeper insight into the inputs, processes and sustainability impacts of organic food before I purchase. |
|  | KN3 | I would prefer to gain substantial information on organic food before I purchase. |
| Attitude (ATT) | ATT1 | I think there is a big difference between organic food consumption and ordinary consumption. |
|  | ATT2 | I think organic food consumption is a very meaningful thing for environmental protection. |
|  | ATT3 | I think organic food consumption is closely related to my life. |
| Subjective Norm (SN) | SN1 | I feel under social pressure to purchase organic food. |
|  | SN2 | People who are important to me think I should purchase organic food. |
|  | SN3 | People who are important to me approve of my organic food purchase. |
| Perceived Behavioral Control (PBC) | PBC1 | Whether or not I purchase organic food is entirely up to me. |
|  | PBC2 | I have the resources, time or opportunity to buy organic food. |
|  | PBC3 | I can easily buy organic food if I want to. |
| Social Media (SM) | SM1 | I use social media to find information about organic food. |
|  | SM2 | I encounter a large amount of information about organic food on social media. |
|  | SM3 | I seek information about organic food from other consumers through online platforms. |
|  | SM4 | Social media increases my awareness and understanding of organic food. |
| Eco-label (EL) | EL1 | I am familiar with the term eco-labeling. |
|  | EL2 | I can recognize the eco-label seal. |
|  | EL3 | Environmental protection-related information is provided on the eco-label package. |
| Government Support (GS) | GS1 | I believe that the government is strongly supporting the development of organic food. |
|  | GS2 | I believe that the government has formulated a strict management system for the authenticity of organic food information. |
| Purchase Intention (PI) | PI1 | I will consider buying organic food. |
|  | PI2 | I am willing to buy organic food instead of conventional food while shopping. |
|  | PI3 | I intend to purchase organic food. |
|  | PI4 | I will make an effort to buy organic food in my next purchase. |

# Appendix 2

| Total Variance Explained | | | | | | |
| --- | --- | --- | --- | --- | --- | --- |
| Initial Eigenvalues | | | Extraction Sums of Squared Loadings | | |  |
| Total | % of Variance | Cumulative % | Total | % of Variance | Cumulative % |  |
| 10.781 | 38.502 | 38.502 | 10.781 | 38.502 | 38.502 |  |
| 2.486 | 8.879 | 47.381 | 2.486 | 8.879 | 47.381 |  |
| 1.769 | 6.317 | 53.698 | 1.769 | 6.317 | 53.698 |  |
| 1.236 | 4.414 | 58.112 | 1.236 | 4.414 | 58.112 |  |
| 1.173 | 4.188 | 62.299 | 1.173 | 4.188 | 62.299 |  |
| .978 | 3.493 | 65.793 |  |  |  |  |
| .857 | 3.060 | 68.853 |  |  |  |  |
| .765 | 2.730 | 71.584 |  |  |  |  |
| .707 | 2.523 | 74.107 |  |  |  |  |
| .634 | 2.266 | 76.373 |  |  |  |  |
| .554 | 1.978 | 78.352 |  |  |  |  |
| .523 | 1.868 | 80.219 |  |  |  |  |
| .501 | 1.791 | 82.010 |  |  |  |  |
| .468 | 1.670 | 83.680 |  |  |  |  |
| .454 | 1.622 | 85.302 |  |  |  |  |
| .426 | 1.522 | 86.824 |  |  |  |  |
| .402 | 1.436 | 88.260 |  |  |  |  |
| .385 | 1.374 | 89.634 |  |  |  |  |
| .378 | 1.350 | 90.984 |  |  |  |  |
| .356 | 1.270 | 92.253 |  |  |  |  |
| .336 | 1.201 | 93.454 |  |  |  |  |
| .330 | 1.178 | 94.632 |  |  |  |  |
| .298 | 1.063 | 95.695 |  |  |  |  |
| .288 | 1.029 | 96.724 |  |  |  |  |
| .252 | .900 | 97.625 |  |  |  |  |
| .238 | .849 | 98.473 |  |  |  |  |
| .233 | .831 | 99.305 |  |  |  |  |
| .195 | .695 | 100.000 |  |  |  |  |
|  | | | | | | |

# Appendix 3

Full-collinearity VIF assessment

| **Construct** | Full-collinearity VIF |
| --- | --- |
| Attitude | 2.124 |
| Eco-label | 2.208 |
| Government support | 1.599 |
| Information seeking | 1.735 |
| Knowledge | 2.310 |
| Perceived behavioral control | 2.241 |
| Purchase intention | 2.229 |
| Social media | 1.900 |
| Subjective norm | 1.521 |
